# Early Assessment of Response to Radiofrequency Ablation With CT Perfusion Imaging in Rabbit VX2 Liver Tumor Model

**DOI:** 10.3389/fonc.2021.728781

**Published:** 2021-11-25

**Authors:** Xiaofei Yue, Xiangjun Dong, Mengting Huang, Hongli Yang, Kun Qian, Changhong Yi, Osamah Alwalid, Yanqiao Ren, Ping Han, Qian Li

**Affiliations:** ^1^ Department of Radiology, Union Hospital, Tongji Medical College, Huazhong University of Science and Technology, Wuhan, China; ^2^ Hubei Province Key Laboratory of Molecular Imaging, Wuhan, China; ^3^ Department of Radiology, The Second Affiliated Hospital of Yangtze University, Jingzhou, China

**Keywords:** radiofrequency ablation, HCC, computed tomography, CT perfusion images, VX2 tumor models

## Abstract

**Objectives:**

To discriminate viable tumors from benign periablational enhancement (BPE) in early stage after radiofrequency ablation (RFA) is a major confounding problem. The goal of this study is to evaluate quantitative assessment and diagnostic value of CT perfusion between viable tumors and BPE after RFA in the rabbit liver VX2 tumor model, with pathological results as the standard.

**Methods:**

Twenty-eight VX2 liver tumors were treated with RFA, on days 1, 3, 7, and 14, seven rabbits were randomly chosen for CT perfusion and performed pathology examinations immediately. The perfusion parameters along with the profile of time-density curves (TDCs) and pseudo-color images of the parameters were observed in both BPE and viable tumors, then compared with the pathology results. The perfusion parameters included blood flow (BF), blood volume (BV), time to peak (TTP), permeability (P), arterial liver perfusion (ALP), portal venous perfusion (PVP) and hepatic perfusion index (HPI).

**Results:**

A total of 26/28 rabbits successfully underwent CT perfusion, while 6/26 lesions were confirmed to be viable tumors. The TDCs of BPE were mainly speed-up platform curves (15/26), while the viable tumors showed mainly speed-up speed-down (3/6) and speed-up platform (2/6) curves. The PVP values were significantly higher, and the HPI values were significantly lower for BPE at all time points than viable tumors (P < 0.05). Both of PVP value and HPI value have high efficiency for the differential diagnosis of the viable tumors and BPE at each time point. These characteristics of CT perfusion parameters were consistent with pathological changes.

**Conclusions:**

The TDCs, PVP and HPI have the potential to indicate BPE and viable tumors effectively early after RFA treatment, the results were highly consistent with pathology. CT perfusion has advantages with great efficacy in monitoring the therapeutic effect early after RFA treatment.

## Introduction

Hepatocellular carcinoma (HCC) is one of the most common malignant tumors in China, and its incidence and mortality rates rank first worldwide. However, less than 30% of patients have the opportunity to undergo surgery when treated in the hospital for a variety of reasons. Therefore, local tumor ablation therapies, especially radiofrequency ablation (RFA), have become the most frequently used alternative treatments for unresectable liver tumors (1–5).

However, local residual and recurrent tumors due to several possible factors can be a main cause of treatment failure (6). Therefore, the appropriate and timely assessment of ablation efficacy is crucial to the success of the therapy. This assessment is usually performed with conventional contrast-enhanced computed tomography (CT) or magnetic resonance imaging (MRI) (1, 2, 7). However, small viable tumors can be concealed by markedly enhanced inflammatory periablational lesions, leading to misdiagnoses in the early period, within one month after onset (6). As confirmed by the use of tissue biopsy controls (8–10), the rates of detecting residual tumors after RFA by CT and MRI are only 36-86%, which encourages the development of functional imaging techniques. Among those choices, fluorine 18 fluorodeoxyglucose positron emission tomography (PET)-CT is the most accurate detection method that directly reflects the functional activity of lesions (11), but it cannot be used clinically as a routine tool because of disadvantages such as the need for special equipment, long scanning times and high cost.

Hepatic CT perfusion (CTP) imaging allows qualitative and quantitative analyses of the microcirculation of the liver by the repetitive sampling of contrast agent uptake in the hepatic parenchyma at a high temporal resolution, reflecting the physiological or pathological status of the liver. Hepatic perfusion parameters, especially the hepatic perfusion index (HPI), have been shown to be good biomarkers for assessing the therapeutic response of hepatic neoplasms in the early stage (within at least 1 month after treatment) (12, 13) and useful for the evaluation of tumor angiogenesis (14, 15).

However, whether CTP can be used to assess the therapeutic effect directly after RFA treatment and its diagnostic value remain unclear. Rabbit VX2 liver tumor model have been widely used in preclinical studies for evaluating anti-tumor response (16–18). Based on the established rabbit liver VX2 RFA treatment model, the purpose of our study was to explore the features and pathological changes in viable tumors and benign periablational enhancement (BPE) using total-liver-volume CTP with the goal of improving the sensitivity and specificity of the early assessment of the response to RFA.

## Materials and Methods

### Animals and VX2 Liver Tumor Model

The animal experiments were performed in accordance with the Guide for the Care and Use of Laboratory Animals and approved by the Institutional Animal Care and Use Committee at Tongji College, Huazhong University of Science and Technology, and all animals received humane treatment throughout the experiment.

A total of 28 adult male Japanese white rabbits weighing 2.5 to 3.0 kg were involved (19, 20). A tumor-bearing rabbit was used to develop the tumor masses for implantation. VX2 tumor cells were successfully implanted into the hind legs of the tumor-bearing rabbit by deep intramuscular injection. Two weeks after inoculation, the tumors were harvested, cut into fragments 1 mm^3^ in size, and placed in Hanks solution for implantation. Twenty-eight recipient Japanese white rabbits were anesthetized with 30-35 mg/kg intravenous sodium pentobarbital. Then, a fresh piece of VX2 tumor tissue was embedded 10 mm deep into the medial left liver lobe of each recipient rabbit, which was exposed through a subxiphoid abdominal incision under strict sterile conditions (21).

### Radiofrequency Ablation Model

Contrast-enhanced CT scans were performed on the 28 rabbits on day 15 after tumor inoculation to evaluate VX2 tumor growth. The implanted tumor masses were controlled within 1.5 cm in diameter. RFA was performed under laparotomy with a RITA 1500X RF system and a 14-G RITA XL multielectrode needle (RITA Medical Systems, Mountain View, CA, USA). The left lobe of the liver with the implanted tumor was exposed through a subxiphoid abdominal incision under sterile conditions. The electrode was inserted into the periphery of the tumor 1.0 cm deep; the expansion radius of the RFA multipolar needle was 1.0 cm, and RFA was performed at 40 W with continuous ablation. The needle tips were heated to 90 ± 5°C and maintained at this temperature for 2.5~5 min, forming ablation lesions of approximately 2.0 cm. Abdominal closure was performed in rigorous hemostatic steps. In addition, the rabbits received active anti-infection treatment and were then housed in hutches. The rabbits were observed after they regained consciousness.

### CT Perfusion Examination

All 28 tumor ablation rabbits were divided into four groups (A, B, C, D) under simple random sampling, with 7 rabbits in each group, and underwent CT perfusion imaging examination on days 1, 3, 7 and 14th after RFA. Each anesthetized rabbit was laid supine and secured onto the rabbit operating table, and respiratory movements were restricted using abdominal bandages.

CT perfusion scanning was then performed with a 128-slice spiral CT system (Siemens SOMATOM Definition AS +; Siemens Medical Solutions, Germany) in the Siemens 4D spiral mode. An initial plain scan was obtained to determine the optimal position for the perfusion scan. Then, the perfusion scan was performed with 6 ml of a contrast agent (300 mg I/ml; Omnipaque, GE Healthcare) injected at a rate of 1.0 ml/s through a 24-G catheter in an ear vein. The perfusion protocol, which was performed every 1.5 s for 60 s, was executed using the following parameters: tube voltage, 100 kV; tube current, 150 mA; matrix, 512 × 512; collimator width, 32 × 1.2 mm; cycle time, 1.5 s; coverage range, 96 mm (including the whole portion of the liver and the upper pole of the right kidney).

### Image Analysis

Image analysis was performed by two observers with 5 years of experience in CT diagnosis. CT volume perfusion scan data from all phases were reconstructed into axial images (3 mm in thickness) and analyzed on the MMWP workstation using analysis-based perfusion software (syngo.via, VPCT Body Perfusion, Siemens). Automatic motion correction and noise reduction algorithms were applied. A region of interest (ROI) was manually placed in the proximal celiac artery and portal vein as the input artery and the inflow vein. The upper pole of the right renal cortex was chosen as a reference for perfusion to measure the arterial and portal venous input according to the maximum slope model (22). The two independent observers performed the perfusion measurements on the maximum-intensity projection (MIP) maps by drawing a freehand ROI around the BPE and viable tumors to obtain the time-density curves (TDCs), as well as the perfusion parameters, including blood flow (BF), blood volume (BV), time to peak (TTP), permeability (P), arterial liver perfusion (ALP), portal vein perfusion (PVP) and hepatic perfusion index (HPI). Pseudocolor perfusion maps were automatically generated, serving for further analysis. Three ROIs were placed on different slices of each targeted area and then used to calculate the average value. ROIs were made as large as possible while avoiding large blood vessels and other surrounding structures to maximize the accuracy of the perfusion values.

### Histopathological Examination

Rabbits were euthanized with air embolism under anesthesia immediately after the CT perfusion scans. The tumors were excised and incised along the direction of the ablation needle. The maximum diameter of the ablation areas was measured, and the samples corresponding to the CTP were fixed with 10% formalin, embedded in paraffin, sectioned at 3μm and stained with hematoxylin and eosin (H&E). The areas of ablation, benign RFA region, viable tumors and surrounding normal liver tissue were investigated by histological findings based on the consensus opinion of two pathologists. Histological sections were observed under an optical microscope (Nikon TE2000, Japan) at 40×, 200×, 400× and digital images were obtained using a camera and microscope system (Nikon DS-U3, Japan).

### Statistical Analysis

The perfusion parameters were presented as the mean ± standard deviation. All analyses were performed using SPSS software 18.0 (SPSS, version 21.0, IBM Corporation, Armonk, NY, USA) and GraphPad Prism software 8.0.2 (GraphPad Software, San Diego, California, USA). Mann-Whitney U-test was used to compare the perfusion parameters between the BPE and viable tumors at different time points. P < 0.05 indicated a statistically significant difference. Receiver operating characteristic (ROC) curves were used to compare the potential performance of diagnostics value of each parameter.

The TDCs of the celiac trunk artery and portal vein were chosen as reference TDCs, and the types of TDCs of BPE and viable tumors at different time points after RFA were analyzed. We categorized TTP curves between those of the celiac trunk and portal vein as speed-up curves, while those later than the curves of the portal vein were categorized as slow-up curves. From the peak time to the end of the scan (60 s), the curve was categorized as a speed-down curve if its trend was closer to the curve of the artery and as a speed-down curve if its trend was closer to the curve of the portal vein. If the downtrend was similar to that of the adjacent normal liver parenchyma, it was categorized as a platform curve.

## Results

### VX2 Tumor and RFA Model

VX2 tumors were successfully grown in the left liver lobe of all 28 rabbits. The tumors ranged from 0.60 cm to 1.50 cm in diameter, and the mean diameter was 1.14 ± 0.35 cm. Across the four experimental groups, with the exception of the death of 2 rabbits in the 14-day group, the remaining 26 tumor-bearing rabbits successfully underwent perfusion CT examination after RFA treatment. There were 26 local ablation lesions, among which 6 viable tumors were confirmed by histopathology. On the 14th day after RFA treatment, we observed partial abdominal wall and intrahepatic metastasis in two rabbits.

### TDCs of BPE and Viable Tumors

**Figure 1:**
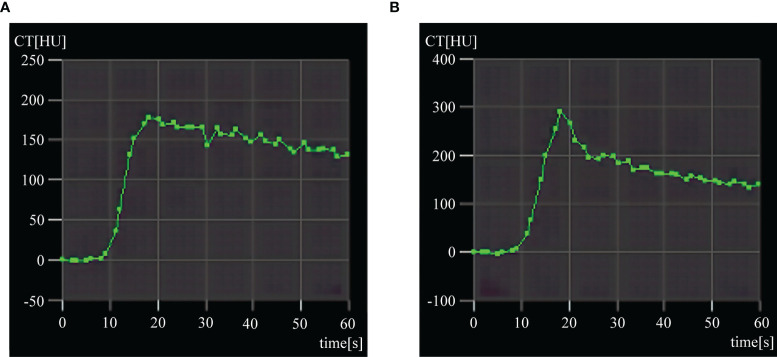
Typical time-density curves (TDCs) of benign periablational enhancement (BPE) and viable tumor. **(A)** Typical speed-up and platform TDC of BPE after 3 days. **(B)** Speed-up and speed-down TDC of residual tumor.

The seven types of TDCs identified in both BPE and viable tumors were shown in **Table 1**. Most of the BPE curves were speed-up platform curves (15/26), followed by speed-up slow-down curves (8/26). Although the peaks of the curves were different, the types of TDCs for BPE were similar in the different time groups. The highest peaks were observed in the 3-day group, and the peaks were significantly reduced in the 14-day group. The curves of the viable tumors comprised speed-up speed-down curves (3/6) and speed-up platform curves (2/6). **Figure 1** provides information on the typical BPE and viable tumor curves.

**Table 1:** Time-density curve (TDC) type.

		Speed-up speed-down	Speed-up slow-down	Speed-up platform	Slow-up speed-down	Slow-up slow-down	Slow-up platform	Platform	Sum
BPE	Day 1	0	1	3	0	2	1	0	7
	Day 3	0	3	4	0	0	0	0	7
	Day 7	0	3	4	0	0	0	0	7
	Day 14	0	1	4	0	0	0	0	5
Viable Tumors		3	1	2	0	0	0	0	6

BPE, Benign periablational enhancement.

### Perfusion Parameters and Corresponding Color Maps in BPE and Viable Tumors

**Figure 2:**
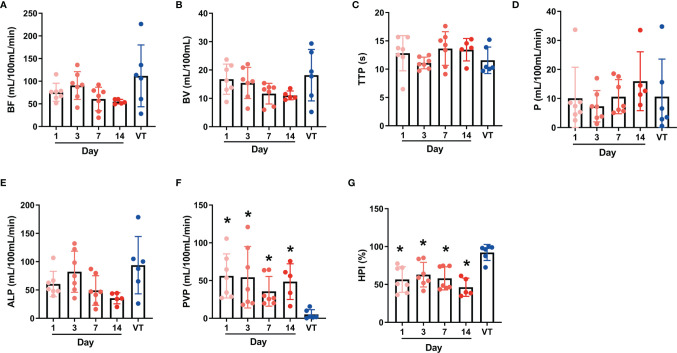
Individual values of postoperative perfusion parameters of benign periablational enhancement (BPE) and viable tumors **(A–G)** represent the individual values of CT perfusion parameters BF, BV, TTP, P, ALP, PVP, and HPI respectively. BPE, Benign periablational enhancement; BF, blood flow; BV, blood volume; TTP, time to peak; P, permeability; ALP, arterial liver perfusion; PVP, portal vein perfusion; HPI, hepatic perfusion index. *P < 0.05, compare with VT (viable tumors).

The statistical results in **Table 2** and **Figure 2** indicated that on 1, 3, 7 and 14 days after RFA treatment, the PVP values were significantly higher for BPE than viable tumors (P < 0.05), whereas the HPI values were significantly lower for BPE than viable tumors (P < 0.05). The values of BF, BV and ALP in viable tumors were increased relative to those of BPE at each time point, but there were no significant differences (P > 0.05). Similarly, there was no significant difference (P > 0.05) in the BF, BV and ALP values among the different time points, although these parameters showed gradual reductions over time. The values of PVP and HPI showed no significant differences among the time points (P > 0.05) and no significant trends.

**Table 2:** Comparison of postoperative perfusion parameters of BPE and viable tumors after RFA treatment.

		BF (mL/100mL/min)	BV (mL/100mL)	TTP (s)	P (mL/100mL/min)	ALP (mL/100mL/min)	PVP (mL/100mL/min)	HPI (%)
BPE	Day 1	75.61 ± 19.84	16.75 ± 5.33	12.82 ± 3.12	10.10 ± 10.65	60.57 ± 22.37	56.13 ± 29.24^*^	56.59 ± 16.86^*^
	Day 3	90.53 ± 30.63	15.42 ± 5.48	11.09 ± 1.03	7.33 ± 5.39	82.24 ± 36.39	54.32 ± 40.69^*^	62.90 ± 16.24^*^
	Day 7	60.73 ± 26.13	11.66 ± 3.64	13.64 ± 2.97	10.63 ± 5.89	49.20 ± 26.09	35.74 ± 19.64^*^	58.09 ± 15.24^*^
	Day 14	54.88 ± 5.09	11.04 ± 1.55	13.44 ± 1.99	15.96 ± 10.13	35.46 ± 9.86	48.55 ± 23.62^*^	46.29 ± 11.98^*^
Viable Tumors		111.86 ± 68.30	18.17 ± 9.07	11.57 ± 2.34	10.66 ± 12.94	93.81 ± 50.57	5.23 ± 6.23	92.20 ± 10.57

BPE, Benign periablational enhancement; BF, blood flow; BV, blood volume; TTP, time to peak; P, permeability; ALP, arterial liver perfusion; PVP, portal vein perfusion; HPI, hepatic perfusion index.

Data are presented as mean ± SD.

*P < 0.05, compare with Viable Tumors.

**Figure 3:**
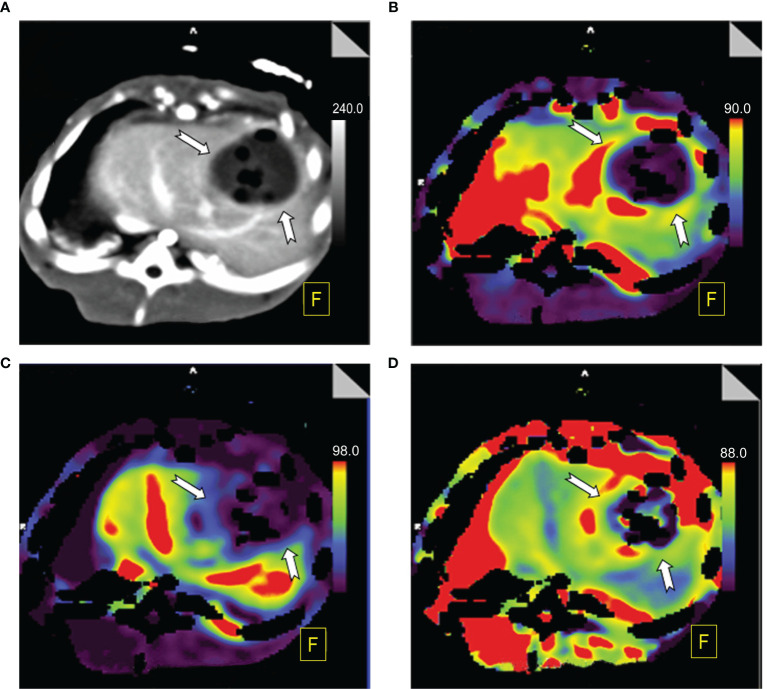
Perfusion color map of benign periablational enhancement (BPE) post-RFA (on day 7). **(A)** Maximum-intensity projection (MIP): The benign periablational enhancement (BPE, white arrow) is the thick, uniform rim surrounding the ablation zone. **(B)** Arterial liver perfusion (ALP): The BPE showed higher perfusion (yellow to red) than the adjacent liver parenchyma, which displayed a wider range of ALP values than shown on MIP images. **(C)** Portal venous perfusion (PVP): The BPE showed lower perfusion (blue). **(D)** Hepatic perfusion index (HPI): The BPE showed higher perfusion (yellow to red).

**Figure 4:**
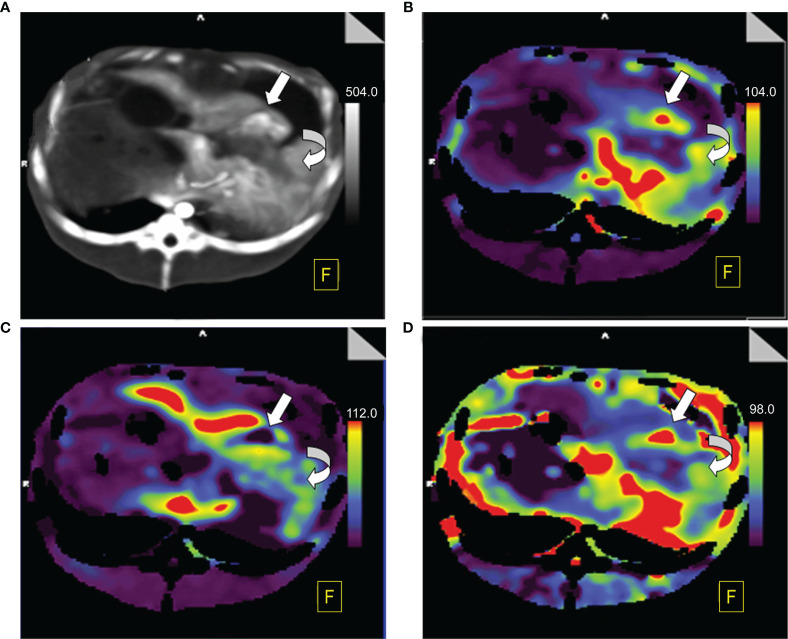
Perfusion color map of viable tumors. **(A)** Maximum-intensity projection (MIP): The viable tumor showed irregular, nodule enhancement around ablation areas (straight white arrow), with a hazy inner margin, and the benign periablational enhancement (BPE, curved white arrow) showed a uniform rim with a hazy outer edge surrounding the ablation zone. **(B)** Arterial liver perfusion (ALP): Viable tumor showed higher perfusion (red) than did the BPE. **(C)** Portal venous perfusion (PVP): Viable tumor showed extremely low perfusion (purple to black) while BPE showed mild to moderate perfusion (blue to green). **(D)** Hepatic perfusion index (HPI): Viable tumor showed significantly higher perfusion (red) than did the BPE (green).

The color maps can reflect significant differences in the ALP, PVP and HPI visually between BPE and viable tumors. On the ALP and HPI maps, BPE showed medium-to-high perfusion regions, whereas viable tumors showed high perfusion. In contrast, regarding the PVP showed hypoperfusion in BPE, while viable tumors showed extreme hypoperfusion. **Figures 3** and **4** show the color maps for BPE (on day 7) and viable tumor perfusion, respectively.

### Diagnostic Performance of the Parameters in BPE and Viable Tumors

**Figure 5:**
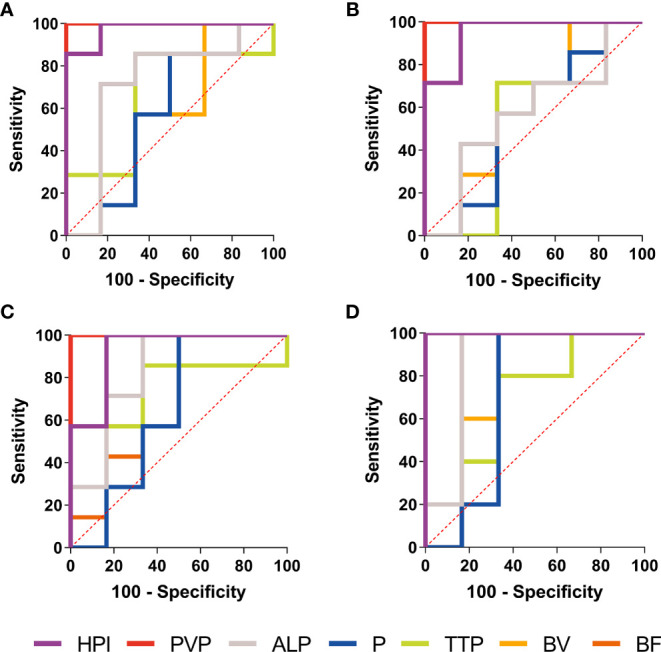
ROC curves of the CT perfusion parameters performance in distinction of BPE and viable tumors. Receiver operating characteristic (ROC) curves of the CT perfusion parameters performance in distinction of BPE and viable tumors in Day 1 **(A)**, Day 3 **(B)**, Day 7 **(C)** and Day 14 **(D)** shown PVP value (red line) and HPI (purple line) had a perfect diagnostic efficiency at each time point.

The diagnostic performance of the parameters in BPE and viable tumors was summarized in **Table 3** and **Figure 5**. ROC curves showed that the PVP value and HPI value had a perfect efficiency for the differential diagnosis of the viable tumors and BPE at each time point. Area under the curve (AUC) of PVP was 1.00 (95%CI: 1.00-1.00) in all time point while AUCs of HPI range 0.93 (0.78-1.00) to 1.00 (1.00-1.00) between different groups. The values of BF, BV, TTP, P and ALP exhibited lower efficiency for the discrimination of the viable tumors and BPE, comparing with PVP and HPI, at each time point, whereas multiple values (BF, BV and ALP) exhibited fair diagnostic efficiency on day 7 and 14, better than their performances on earlier time points.

**Table 3:** Diagnostic performance of each parameter of CT perfusion in distinction of viable tumor and BPE.

Parameter	Day	AUC (95%CI)	Youden index	Sensitivity	Specificity	Critical value
BF (mL/100mL/min)	1	0.643 (0.293 - 0.992)	0.52	85.71	66.67	92.45
	3	0.571 (0.227 - 0.915)	0.38	71.43	66.67	101.10
	7	0.762 (0.463 - 1.000)	0.67	100.00	66.67	99.26
	14	0.833 (0.535 - 1.000)	0.83	100.00	83.33	60.60
BV (mL/100mL)	1	0.548 (0.202 - 0.894)	0.33	100.00	33.33	24.98
	3	0.619 (0.284 - 0.954)	0.38	71.43	66.67	17.25
	7	0.714 (0.376 - 1.000)	0.67	100.00	66.67	16.58
	14	0.767 (0.453 - 1.000)	0.67	100.00	66.67	15.66
TTP (s)	1	0.667 (0.345 - 0.989)	0.52	85.71	66.67	11.27
	3	0.548 (0.191 - 0.904)	0.38	71.43	66.67	10.60
	7	0.714 (0.413 - 1.000)	0.52	85.71	66.67	10.41
	14	0.700 (0.377 - 1.000)	0.46	80.00	66.67	11.94
P (mL/100mL/min)	1	0.571 (0.227 - 0.916)	0.36	85.71	50.00	4.03
	3	0.548 (0.207 - 0.889)	0.23	57.14	66.67	6.87
	7	0.643 (0.304 - 0.982)	0.50	100.00	50.00	4.31
	14	0.700 (0.348 - 1.000)	0.67	100.00	66.67	7.17
ALP (mL/100mL/min)	1	0.714 (0.394 - 1.000)	0.55	71.43	83.33	64.02
	3	0.571 (0.239 - 0.904)	0.26	42.86	83.33	63.71
	7	0.833 (0.591 - 1.000)	0.67	100.00	66.67	87.37
	14	0.867 (0.615 - 1.000)	0.83	100.00	83.33	54.82
PVP (mL/100mL/min)	1	1.000 (1.000 - 1.000)	1.00	100.00	100.00	21.39
	3	1.000 (1.000 - 1.000)	1.00	100.00	100.00	16.83
	7	1.000 (1.000 - 1.000)	1.00	100.00	100.00	17.76
	14	1.000 (1.000 - 1.000)	1.00	100.00	100.00	15.88
HPI (%)	1	0.976 (0.904 - 1.000)	0.86	85.71	100.00	72.37
	3	0.952 (0.839 - 1.000)	0.83	100.00	83.33	86.34
	7	0.929 (0.776 - 1.000)	0.83	100.00	83.33	81.14
	14	1.000 (1.000 - 1.000)	1.00	100.00	100.00	66.93

AUC, area under the curve; CI, confidence interval; BPE, benign periablational enhancement; BF, blood flow; BV, blood volume; TTP, time to peak; P, permeability; ALP, arterial liver perfusion; PVP, portal vein perfusion; HPI, hepatic perfusion index.

### Histopathological Results

**Figure 6:**
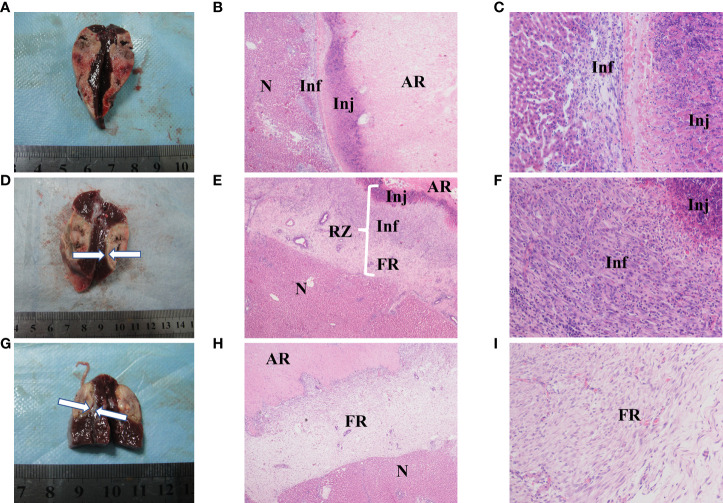
Gross morphology and histological images of benign radiofrequency ablation (RFA) region. The areas represented from peripherial to central ablation are as follows: normal hepatocyte (N); reaction zone (RZ); ablation region (AR). **(A–C)** (on day 3 after RFA): Ablation lesion was shown as well circumscribed, gray-white tissue in gross morphology observation **(A)**, in which AR and RZ cannot be distinguished obviously. 40× H&E histology **(B)** showed AH was dominated by coagulation necrosis. RZ was observed at the periphery of AR, mainly consisted by injury reaction (Inj) and inflammatory (Inf). 200× H&E histology **(C)** showed that Inj was mainly composed of hepatocytes with pyknosis and karyorrhexis, and Inf was manifested as small amount of inflammatory cell infiltration. **(D–F)** (on day 7 after RFA): Gross morphology image **(D)**: granulation tissue around the ablation lesion can be observed (white arrows); 40× **(E)** H&E histology showed the change of RZ, in which Inj shrank while the inflammatory (Inf) and fibrosis increase, and a fibrous rim (FR) had formed in periphery. **(F)** showed Inj and Inf in 200× H&E histology. **(G–I)** (on day 14 after RFA): Gross morphology image of day 14 after RFA **(G)**: granulation tissue around the ablation lesion had gotten thicker and denser (white arrows); 40× **(H)** and 200× **(I)** H&E histology showed Inj and Inf were significantly reduced and even disappeared in some area, RZ was mainly composed by a further thickened FR.

**Figure 7:**
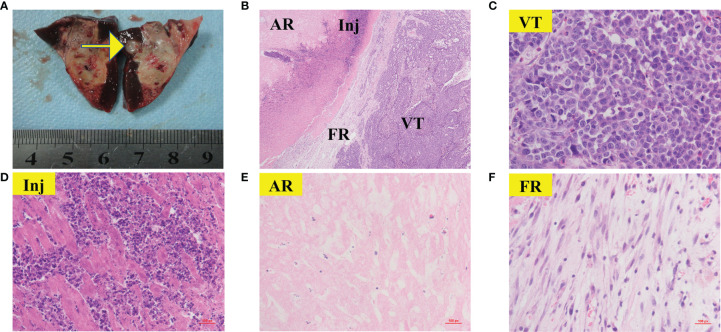
Gross morphology and histological images of viable tumor. The areas represented from periphery to center are as follows: viable tumors (VT); reaction zone (RZ); ablation region (AR) Gross morphology image of viable tumor (**A**, yellow arrow) was appeared as nodular located on the periphery of the RFA region. 40× H&E histology **(B)** showed VT can infiltrate and compress the fibrous rim, causing local RZ thinner than the adjacent tumor-free area. **(C–F)** showed 400× H&E histology of VT**(C)**, Inj **(D)**, AR **(E)** and FR **(F)** from **(B)**. **(C)** showed typical clumps of heteromorphic tumor cells with large, hyperchromatic nuclei.

Rabbits in each group were sacrificed after CTP scanning and histopathological examinations were performed sequentially. Viable tumors were found in 6 of the 26 lesions, which appeared as pale, rubbery nodular tissues located at the periphery of the RFA region in gross morphology observation. Histological examination of H&E showed that the ablation region which located in the center was dominated by coagulative necrosis. A 1-5 mm reaction zone (RZ) was observed at the periphery of ablation region, which consists of three parts: injury, inflammatory cell infiltration and fibrosis, showing transitional changes without clear boundaries. on the 3rd day of ablation, the RZ was dominated by injury reaction, which consisted by the damaged and degenerated cells. Different from the complete coagulation necrosis in ablation region, due to the relatively low temperature, the injury reaction was manifested as pyknosis and karyorrhexis, distributed in bands in the ablation marginal area. Around the injury reaction, there was small amount of inflammatory cell infiltration scattered with slight fibrosis. On the 7th day, the injury reaction shrank (absorption or dissolution) while the inflammatory and fibrosis increase, gradually forming a fibrous rim. On the 14th day, the injury reaction was further significantly reduced and even disappeared in some area, scattered with a few inflammatory cells. The reaction zone was mainly repaired by fibrous tissue, showing as a further thickened fibrous rim. Viable tumors were usually located at the edge of the ablation region. Due to the irregular shape of the tumor, the local temperature was too low to achieve the purpose of ablation. Those viable tumors can infiltrate and compress the fibrous rim, causing part of the fibrous tissue to become thinner than the adjacent tumor-free area. **Figure 6** presents the gross morphology images and high magnification H&E staining findings of the benign RFA region on days 3, 7 and 14 after RFA treatment, and **Figure 7** showed the gross morphological images and high-magnification H&E staining findings of viable tumor.

## Discussion

Contrast-enhanced CT fails to provide desirable sensitivity because the presence of BPE obscures small viable tumors in the early stage. This study applied CT perfusion for the early post-RFA follow-up examination of rabbit VX2 tumors. The results reveal that compared with BPE, viable tumors had a significantly lower PVP and a significantly higher HPI (P < 0.05) at all time points, indicating that compared with BPE, viable tumors had significantly increased hepatic arterial perfusion and decreased or absent portal venous perfusion. The BF, BV, ALP indexes were higher for viable tumors than BPE but without a significant difference (P > 0.05), which indicates that while viable tumors have a richer blood supply than BPE, the difference is not sufficient to reliably differentiate between the two. Considering the pathological findings, the assumptions responsible for these differences are as follows:

1. Heat damage led to increased local BF and abnormal vascular access opening, causing an increase in the blood supply to both BPE and viable tumors. These factors might have greater effects on viable tumors because the tumor vessels lack the normal smooth muscle and pericyte structure to modulate BF (16).
2. The size of viable tumors is relatively small, and they are less necrotic and have a higher microvascular density (MVD), which is related to high activity and a rich blood supply, especially the arterial supply. On the other hand, the intensive microvascular networks and relatively little stroma of viable tumors contribute to the faster intravascular and interstitial contrast equilibration. Hence, the enhancement declines rapidly. By studying changes in the blood perfusion of rabbit liver VX2 tumors at different time points during the tumor growth cycle, Hanping Wu et al. concluded that tumor enhancement in the early phase (day 7 and 14) was more intense and faster and that the clearance of contrast agents was faster compared to tumors enhancement in the late phase (day 21 and 28) (23). Stewart et al. also drew a similar conclusion, which indicates that the stage of tumor development is a factor of its enhancement pattern (24) The results of the TDCs in our study show that half of the curves of viable tumors were speed-up speed-down curves (3/6), and all of these curves were observed in the early stage (day 3 and 7), which is consistent with the pathological findings as well as the findings of previous studies.
3. The postoperative presence of residual tumors following RFA may cause a series of complex changes in endocrine and immune functions in the local tumor microenvironment or the entire body. This may cause a significant increase in tumor angiogenesis, contributing to the increases in the ALP and HPI. Related studies have shown that similar to primary HCC, viable tumors have a high blood supply, which possibly reflects the process of dissimilation in vessels (25, 26). Some studies on biochemical changes in tumor cells have shown that high hypoxia-inducible factor 1α (HIF-1α) and vascular endothelial growth factor A (VEGF-A) expression in viable tumors is conducive to tumor angiogenesis (27, 28). The above observations provide evidence in terms of pathology and molecular biology supporting the current findings that the CTP indexes ALP and HPI were increased significantly and PVP was markedly reduced in viable tumors, providing a functional basis for evaluating tumor prognosis.

Perfusion pseudocolor images not only show perfusion parameters intuitively, with clear positions, but are also not limited by ROI, aiding the detection of small occult lesions. As demonstrated in **Figures 3** and **4**, the ALP, HPI and PVP values of BPE and viable tumors presented in the pseudocolor images are highly consistent with the statistical results. In addition, compared with TDCs and perfusion indexes, pseudocolor images have an advantage in that they can reflect objective changes in the overall perfusion state, unrestricted with respect to the ROI. As shown in **Figure 3**, on day 7, BPE showed higher ALP and HPI and lower PVP perfusion areas that were wider than the enhancement regions observed in the original image. This indicates changes in the partial hemodynamics of the inflammatory response as well as the influence of the surrounding liver parenchyma. These changes may not be clearly demonstrated by pathomorphology; however, perfusion pseudocolor images can show these functional changes in an intuitive manner. In the early period after the treatment of colorectal cancer liver metastases with RFA, Meijerink et al. performed a sensitivity study comparing CT perfusion and PET-CT for the detection of residual tumors (29). Based on pseudocolor images of the hepatic artery, they found that the probability of a liver parenchyma region with abnormal increases in perfusion progresses toward new metastatic lesions was significantly greater than the probability of it retaining normal perfusion. Mahnken et al. conducted a related study and drew similar conclusions (30). These findings indicate that pseudocolor images can provide clear information for determining the range of lesions. They have some sensitivity for detecting changes in the functional status of small lesions, while morphological changes are not obvious in these images.

Although CT perfusion has not been clinically applied due to its high radiation dose, the current technological development in CT is moving towards high speed, low dose, deep learning, and multienergy (31). Low-dose CT perfusion scanning can already be achieved (32–34). Therefore, CT perfusion can be used to evaluate the treatment effect in the early stage without increasing the radiation dose of patients. In the next study, we will also study the application of low-dose CT perfusion in patients with HCC after RFA.

The present study has limitations. First, RFA was used to attempt complete tumor ablation in our study, thus, there were not enough cases of viable tumors for statistical comparison with BPE at the same time point. More detailed studies with larger sample sizes based on a partial RFA model will be carried out in the future. Second, the pathological characteristics of VX2 tumors might be different from those of human HCC tumors because they are derived from a virus-induced papilloma of rabbits.

## Conclusions

In conclusion, CT perfusion has advantages in monitoring the therapeutic response early after RFA treatment, with great potential diagnostic efficacy in distinguishing viable tumors from BPE using the PVP and HPI as well as the TDCs.

## Data Availability Statement

The original contributions presented in the study are included in the article/supplementary material. Further inquiries can be directed to the corresponding authors.

## Ethics Statement

The animal study was reviewed and approved by Institutional Animal Care and Use Committee at Tongji College, Huazhong University of Science and Technology.

## Author Contributions

QL and PH developed the concept and designed the experiments. XY, XD, and MH performed the experiments. HY, KQ, and CY collected and analyzed data. XY and QL wrote the manuscript. OA and YR revised the manuscript. All authors contributed to the article and approved the submitted version.

## Funding

This research was supported by National Natural Science Foundation of China (81371661).

## Conflict of Interest

The authors declare that the research was conducted in the absence of any commercial or financial relationships that could be construed as a potential conflict of interest.

## Publisher’s Note

All claims expressed in this article are solely those of the authors and do not necessarily represent those of their affiliated organizations, or those of the publisher, the editors and the reviewers. Any product that may be evaluated in this article, or claim that may be made by its manufacturer, is not guaranteed or endorsed by the publisher.
